# Novel Use of the ŌNŌ Retrieval System for Intracardiac Thrombectomy in a High-Risk Pediatric Patient

**DOI:** 10.1016/j.jscai.2023.100976

**Published:** 2023-05-19

**Authors:** Aravinth Karunanandaa, Neil Patel, Pierre Wong, Clementine Vo, Michael Van Tienderen, Winston Huh, Emma Cantor, Travis Piester, John Cleveland, Darren P. Berman

**Affiliations:** Children’s Hospital Los Angeles, Los Angeles, California

**Keywords:** percutaneous retrieval, right atrial thrombus, thrombectomy

Optimal management for right atrial thrombus (RAT) lacks consensus agreement.^1^^,^^2^ We describe percutaneous retrieval of a RAT utilizing the ŌNŌ (ŌNŌCOR) retrieval system (Figure 1A, B).

**Figure 1:**
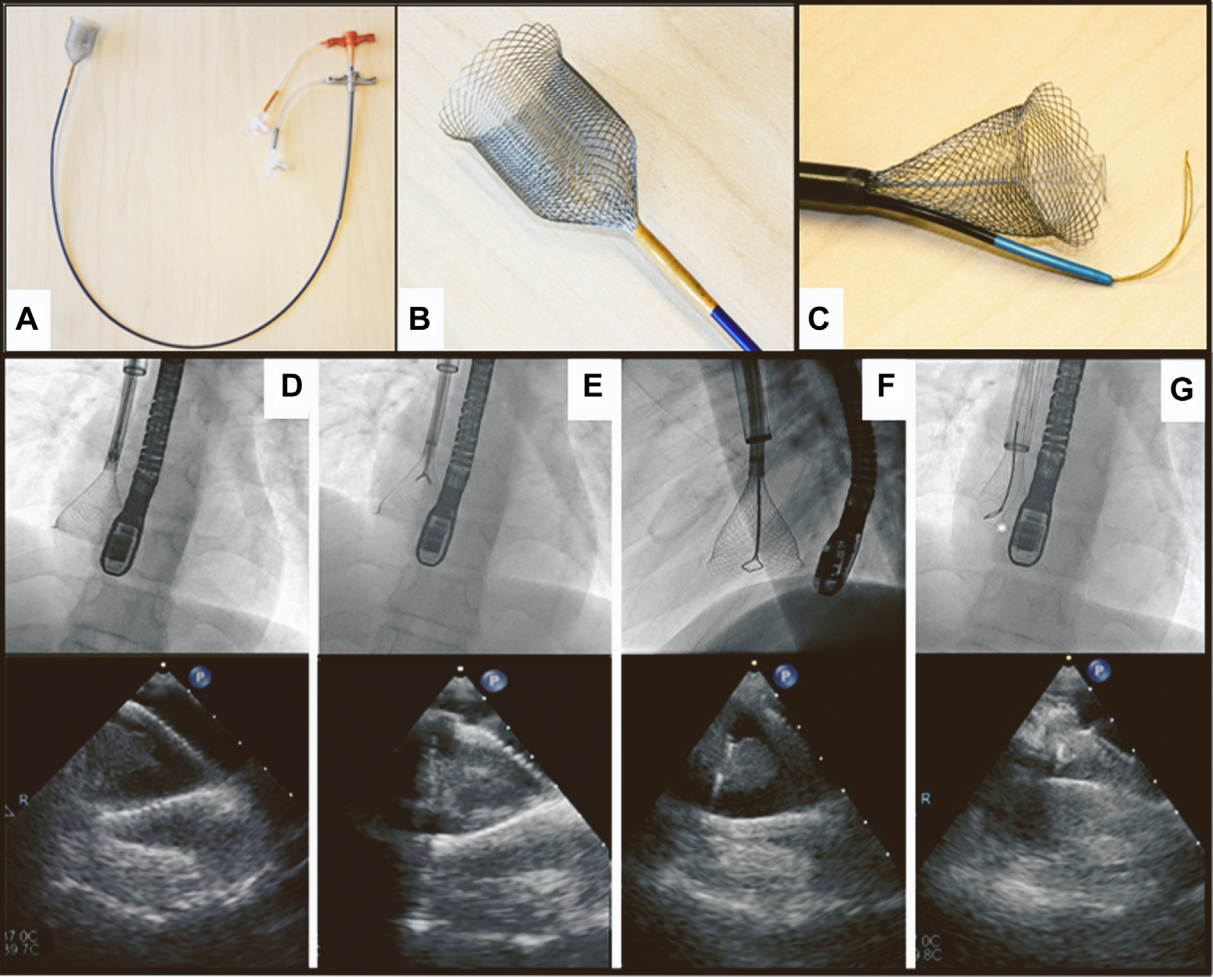
**ŌNŌCOR retrieval system and extraction.** The ŌNŌ is a 12F stainless steel reinforced catheter (**A**) through which a braided nitinol retrieval basket is passed (**B**). The Simplified Extraction of Atrial Tumor with Targeted Loop Electricity (SEATTLE) technique is demonstrated with a snare advanced through the ŌNŌ lumen and the additional electrocautery snare external to the nitinol basket (**C**). On the first attempt, the mass was safely encased by the basket (**D**), and rescue forceps were used to grasp the right atrial mass (**E**). On the second attempt, a 20-mm loop snare was advanced through the ŌNŌ and secured around the mass (**F**). Gentle tension was applied, and the preloaded 27-mm cautery snare was advanced exterior to the basket and secured around the base of the mass. Cautery was delivered as the mass is withdrawn (**G**).

## Case report

A 14-year-old boy with tibial osteosarcoma was found to have a 35 × 13 mm right atrial mass on echocardiogram suspected to be a subacute thrombus given the low likelihood of cardiac metastasis. He was discussed and referred for percutaneous retrieval prior to considering surgical embolectomy.

Transesophageal echocardiography (TEE) demonstrated that the mass was attached to the right atrial floor and not disrupting tricuspid valve motion, suggesting the best catheter trajectory would be from the right internal jugular vein. After serial dilations, a 26F DrySeal sheath (GORE) was placed. Next, a 12F Destino deflectable sheath (OSCOR Inc) was coaxially advanced. Prior to advancing it through the DrySeal, a medium oval-flexible 27-mm polypectomy snare with electrocautery capability (Boston Scientific) was secured next to and around the Destino sheath. The ŌNŌ retrieval system was loaded through the 12F Destino sheath. Under fluoroscopy and X-plane TEE, the ŌNŌ retrieval basket was positioned over the RAT (Figure 1D). A 7F rat tooth/alligator grasping forceps with 8-mm jaw (Boston Scientific) was passed through the ŌNŌ to grasp the RAT (Figure 1E). The ŌNŌ, Destino sheath, and DrySeal sheath were advanced over the entrapped mass as the retrieval forceps were simultaneously pulled, removing a portion of the mass.

To remove the remaining portion of the mass, this approach was repeated using a 20-mm loop snare inserted through the ŌNŌ inner lumen instead of the grasping forceps (Figure 1F). Once the mass was snared and within the ŌNŌ basket, the preloaded 27-mm cautery snare was loosened from the Destino sheath, advanced around the nitinol basket, and secured around the stalk of the remaining mass (Figure 1G). With the basket securely over the mass and with gentle tension on both snares, electrocautery was delivered through the 27-mm snare as it was simultaneously tightened. The mass detached from the right atrial wall and was withdrawn (Supplemental Video 1). TEE demonstrated no residual mass. There were no procedural complications, and the patient was discharged home the following day. The final pathology confirmed an organized thrombus.

## Discussion

We describe the novel use of the ŌNŌ system to retrieve a RAT in a high-risk pediatric patient. The ŌNŌ requires, at minimum, a 12F delivery sheath. In this case, a 26F sheath was utilized, given the uncertainty of the compressibility of the right atrial mass within the ŌNŌ catheter and the desire to use a triaxial dual snare technique. We believe a smaller delivery sheath may be utilized in future cases.

The snare technique utilized in this case (depicted in Figure 1C), designated Simplified Extraction of Atrial Tumor with Targeted Loop Extraction (SEATTLE), was previously described to retrieve an atrial myxoma.^3^ In our case, upon retrieval from the body, the 27-mm cautery snare was still encircled around the base of the mass, suggesting that the combination of cautery and tension on both snares ultimately contributed to the ease in removal. X-plane TEE guidance is recommended to safely snare and cauterize the mass away from the atrial wall.

## Conclusion

This is the first reported pediatric case using the ŌNŌ retrieval system for the purpose of thrombectomy to remove an atrial thrombus and may offer an alternative therapeutic option to surgical embolectomy.

## Supplementary material

Supplemental Video 1 **Second attempt at thrombus retrieval.** On the second attempt, a 20-mm loop snare was advanced through the ŌNŌ and secured around the mass. Gentle tension was applied, and the preloaded 27-mm cautery snare was advanced exterior to the basket and secured around the base of the mass. Cautery was delivered as the mass is withdrawn.
